# Associations between clinical characteristics and tumor response to neoadjuvant chemoradiotherapy in rectal cancer

**DOI:** 10.1002/cam4.4051

**Published:** 2021-06-15

**Authors:** Xiaolin Pang, Yuanhong Gao, Hanchen Yi, Hailing Liu, Shuai Liu, Jian Zheng

**Affiliations:** ^1^ Department of Radiation Oncology The Sixth Affiliated Hospital of Sun Yat‐Sen University Guangzhou China; ^2^ Department of Radiation Oncology Sun Yat‐Sen University Cancer Center Guangzhou China; ^3^ Department of Pathology The Sixth Affiliated Hospital of Sun Yat‐Sen University Guangzhou China; ^4^ Guangdong Provincial Key Laboratory of Colorectal and Pelvic Floor Diseases The Sixth Affiliated Hospital of Sun Yat‐Sen University Guangzhou People’s Republic of China

**Keywords:** adjuvant chemotherapy, good pathological response, locally advanced rectal cancer, neoadjuvant chemoradiotherapy, risk factors

## Abstract

Following standard neoadjuvant chemoradiotherapy and total mesorectal excision, some patients with locally advanced rectal cancer achieve good response (pathological T0–2N0), while others show nonresponse (pathological T3–4N0 or node‐positive). To date, the clinicopathological predictors of good response and the necessity of adjuvant chemotherapy treatment (ACT) in good responders remain unclear. In this retrospective study, clinicopathological characteristics were surveyed to investigate the correlation with good response; furthermore, a propensity score matching (PSM) model was designed to balance the confounding factors between good responders treated with ACT or observation. A total of 2255 patients were enrolled, including 1069 good responders and 1186 nonresponders. The results of the survival analysis showed a good response predicted a better 3‐year prognosis (*p* < 0.001). The logistic regression analysis showed less advanced T and N stages (T3 vs. T4; N0 vs. N1–2), more neoadjuvant chemotherapy (nCT) cycles (≥4 vs. 1–3), and delayed surgery (≥8 weeks vs. <8 weeks) were independent predictors of a good response (*p* < 0.05). Especially, patients treated with both more nCT cycles and a delay in surgery included the greatest number of good responders (*p* < 0.001). For good responders, after PSM (1:3), 235 observation cases were matched to 705 ACT cases. As compared with observation, ACT had no greater impact on prognosis analysis (*p* > 0.05). In conclusion, more cycles of nCT and a delay in surgery predicted a better response, and the delivery of ACT might be omitted in good responders.

## Graphical abstract

Our study demonstrated that good response patients (pathological T0–2N0) to neoadjuvant chemoradiotherapy (nCRT) in a locally advanced rectal cancer population experienced significantly better 3‐year prognosis than nonresponsive patients (pathological T3–4N0 or node‐positive). Our results also showed that less advanced cT and cN stages, more neoadjuvant chemotherapy cycles (≥4), and delayed surgery (≥8 weeks) were significantly associated with a good response to nCRT. Especially, the combination of more neoadjuvant chemotherapy cycles and a delay in surgery achieved the highest proportion of good responses. Further analysis demonstrated that adjuvant chemotherapy in the good responders was not recommended.

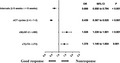

## 1 INTRODUCTION

Preoperative chemoradiotherapy followed by total mesorectal excision (TME) surgery has become the standard treatment for locally advanced rectal cancer (LARC).^1^ Following radical surgery, it was reported that 14%–27.5% of patients achieve pathologic complete response (pCR)^2^, ^3^; and some patients were downstaged to pathological stage I (ypI: ypT1–2N0M0), while other patients experienced no or little response (ypT3–4N0M0 or node‐positive).^3^, ^4^, ^5^, ^6^ Accumulated studies have confirmed pathological good responses, such as pCR and ypI, are associated with good overall survival (OS) and disease‐free survival (DFS) rates.^3^, ^5^, ^7^, ^8^ Furthermore, over the past two decades, the use of organ preservation strategies such as ‘wait‐and‐watch’ and local excision in patients with a good response to neoadjuvant chemoradiotherapy (nCRT) is becoming increasingly popular.^6^, ^9^, ^10^ Thus, the interest in investigating the primary clinicopathological tumor characteristics and treatment strategies that predict a pathological good response is growing, since these aspects may influence the choices of preoperative treatments and subsequent management techniques in the future.^8^, ^11^, ^12^, ^13^

Several prospective clinical trials have investigated the scheduling of nCRT.^14^, ^15^, ^16^, ^17^ Gao et al. suggested that the sandwich treatment approach, involving induction, concurrent, and consolidation chemotherapy, would result in a 42.2% of pCR rate and 82.2% of downstaging.^16^ A clinical trial from Memorial Sloan Kettering Cancer Center (MSKCC) reported that delivering consolation chemotherapy and lengthening the interval between radiation and surgery increased the proportion of pCR.^15^ Recently, the RAPIDO study reported that a delay in surgery after short‐course radiotherapy may achieve a greater proportion of pCR.^14^ Similarly, some studies have validated the clinical T stage, baseline serum carcinoembryonic antigen (CEA) level, kirsten rat sarcoma viral oncogene (KRAS) mutation status, tumor height, and magnetic resonance imaging‐based extramural venous invasion (MRI‐EMVI) status would be applied as predictors of a pathological good response.^8^, ^12^, ^13^, ^18^ However, published studies have mainly focused on either the pathological characteristics or the modified treatment regimens and schedules^12^, ^13^, ^18^; few investigations to date have combined both to predict a pathological response.^8^, ^11^

Furthermore, among patients who downstaged to a pathological good response, the necessity of adjuvant chemotherapy treatment (ACT) was also deemed controversial.^5^, ^7^, ^19^, ^20^, ^21^, ^22^ Several studies have reported that no survival benefits exist in concert with ACT among these good responders.^20^, ^22^ Nevertheless, other studies have shown that the delivery of ACT achieved an improvement in the DFS, thus supporting the necessity of ACT.^5^, ^19^, ^21^ However, these studies were often either small in scale or did not consider the preoperative clinical characteristics, limiting their ability to draw valid conclusions.^5^, ^7^, ^19^, ^21^, ^22^

Here, we undertook a large‐scale retrospective analysis of all consecutive rectal cancer patients treated by nCRT followed by TME surgery to exploring the correlation between clinicopathological characteristics, treatment regimens, and the achievement of a pathological good response (pCR and ypI). Furthermore, we conducted a propensity score matching (PSM) analysis to balance the preoperative tumor characteristics and explore the necessity of ACT for the pathological good responders.

## 2 MATERIALS AND METHODS

### 2.1 Patients

Patients with pathological confirmed rectal cancer who underwent nCRT followed by TME surgery were identified from the database of the Sixth Affiliated Hospital of Sun Yat‐sen University and Sun Yat‐sen University Cancer Center from December 2007 to January 2019. Inclusion criteria were the existence of a pathological defined adenocarcinoma of clinical stage II or III (T3‐4N0‐2M0) located within 10 cm of the anal verge; a history of at least one cycle of neoadjuvant chemotherapy (nCT) being delivered; available data of clinicopathological characteristics; and the cycles and regimens of treatment being complete. Exclusion criteria included the existence of a primary tumor with distant metastasis and surgery with local or palliative excision without full consideration. In order to reduce the bias caused by serious postoperative complications, patient who died within 60 days from operation were also excluded. This study was approved by the ethics committee of the Sixth Affiliated Hospital of Sun Yat‐sen University.

### 2.2 Treatment

The treatment decisions were comprehensively discussed by a multidisciplinary team (MDT). All included patients received nCRT followed by TME surgery. Regarding the radiotherapy protocol, most patients were subjected to intensity‐modulated radiotherapy treatment (IMRT), while a few patients underwent three‐dimensional conformal radiotherapy treatment (3D‐CRT). In brief, a total of 45 and 50 Gy for 25 fractions were administered to the gross tumor and entire pelvic area, respectively.^23^ All patients received concurrent chemotherapy for at least one cycle. Moreover, a subgroup of patients was given induction or consolidation chemotherapy or both. The regimens were fluorouracil‐based FOLFOX or CAPOX.^24^ The chemotherapy tolerance and clinicopathological characteristics such as age, clinical TNM staging, and tumor differentiation were mainly determining the factors of the cycles and the regimens of the nCT. Following the completion of neoadjuvant treatment, all patients underwent TME surgery. The waiting time from the collection of data concerning the last fraction of radiation to TME surgery was recorded and, when the period was more than 8 weeks, the case was defined as having undergone delayed surgery. All radical surgeries were implemented by surgeons who had been trained in gastrointestinal department for more than 10 years. After TME surgery, patients with pCR or stage ypI were defined as good responders; otherwise, patients with ypT3–4N0M0 or N‐positive outcomes were considered to be nonresponders.^5^, ^8^ The decisions of whether adjuvant chemotherapy was delivered or not, the number of cycles, and the regimens used were based on discussion among members of the MDT.

### 2.3 Follow‐up

After TME surgery, all patients were followed up with according to the National Comprehensive Cancer Network guidelines as follows: chest and abdomen computed tomography, contrast‐enhanced pelvic magnetic resonance imaging, and tumor biomarker tests were performed at 3‐month intervals for the first 2 years, at 6‐month intervals for the next 3 years, and 1‐year intervals thereafter.^24^ Cancer‐specific survival (CSS) was defined as the time from the date of surgery to death caused by tumor progression or, when censored, at the latest follow‐up date if the patient was still alive. Locoregional relapse‐free survival (LRFS) and distant metastasis‐free survival (DMFS) were defined as the time from surgery to the date of local recurrence or distant metastasis, respectively, or to the date of death or, when censored, the latest date of follow‐up. DFS was defined as the time from the date of surgery to the date of disease relapse or death, or, when censored, the latest date of follow‐up.

### 2.4 Statistical analysis

Inter‐group Pearson's chi‐squared tests were performed to analyze the clinical factors associated with good response. A logistic regression analysis with a likelihood ratio stepwise approach was applied to identify the significant clinical factors associated with a good response. After surgery, patients who were staged pathological as T0–2N0M0 receiving adjuvant chemotherapy were enrolled in the ACT group; otherwise, they were enrolled in the observation (OB) group. PSM was performed for both groups. The matching ratio was 1:3 and the covariates included age, sex (male vs. female), tumor differentiation (poorly, moderately, or well differentiated), baseline clinical T and N stages, clinical TNM stages, radiation technique (3D‐CRT vs. IMRT), number of cycles of nCT (1–3 vs. ≥4), and the time to surgery (<8 weeks vs. ≥8 weeks). Survival analyses of the ACT and OB groups were performed using the Kaplan–Meier product‐limit method; similarly, this same method was adopted to analyze the survival differences between the good response and nonresponse groups. The whole procedure of statistical analysis was performed using the Statistical Package for the Social Sciences version 26.0 software program (IBM Corporation). A difference with a two‐sided *p* value of <0.05 was considered to be statistically significant.

## 3 RESULTS

### 3.1 Patients and clinicopathological characteristics

**FIGURE 1:**
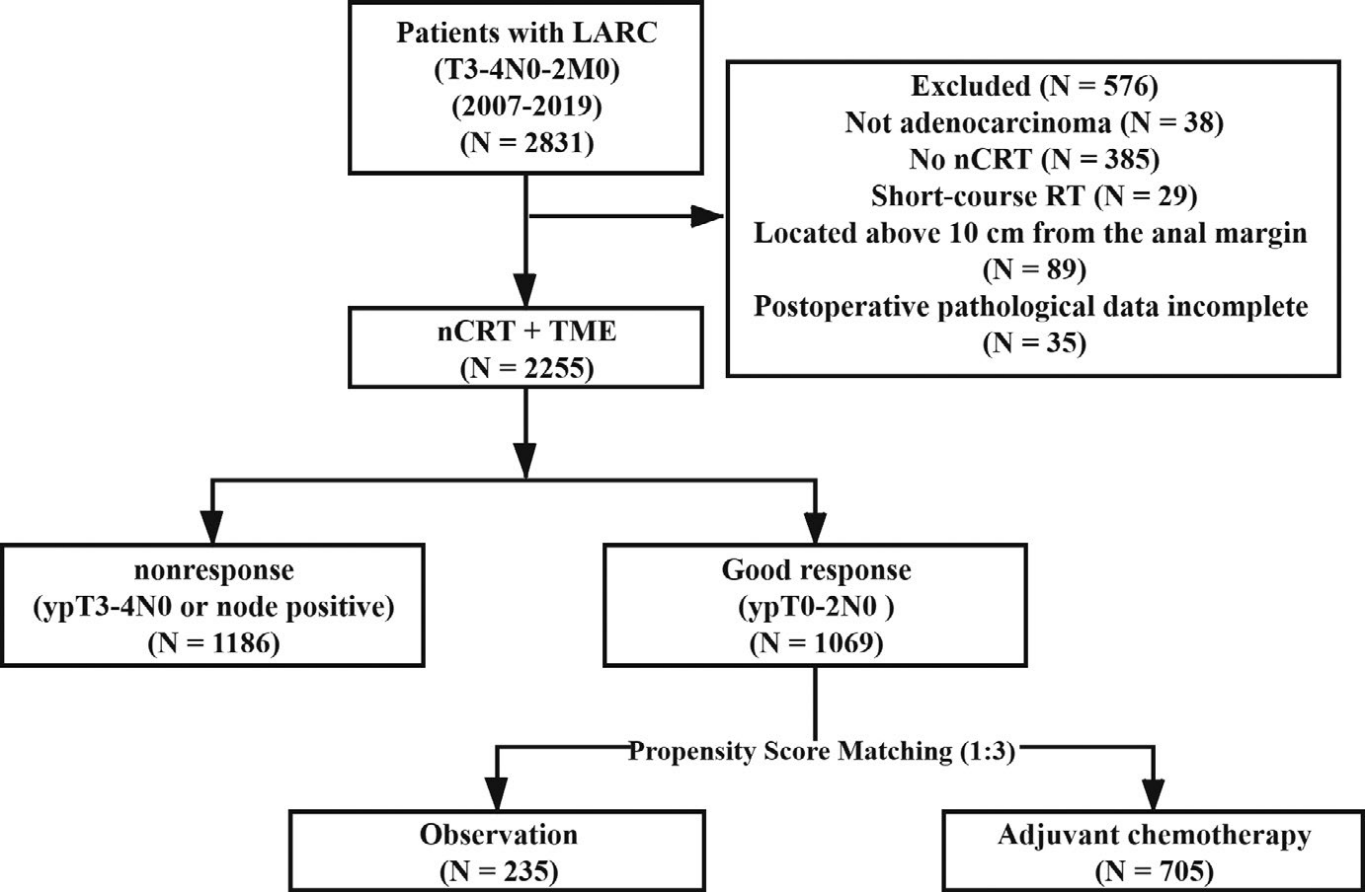
Patient flowchart. Abbreviations: LARC, locally advanced rectal cancer; nCRT, neoadjuvant chemoradiotherapy; RT, radiation therapy; TME, total mesorectal excision

Overall, 2255 patients who underwent nCRT followed by curative TME surgery were included. Of these, 1069 (47.4%) patients were responsive and 1186 (52.6%) patients were nonresponsive (Figure 1). The median age of the study population was 56 years (range: 19–80 years) and the median follow‐up time was 40 months (range: 9–149 months). The clinicopathological characteristics of the two groups were summarized in Table 1.

**TABLE 1:** Preoperative clinicopathological characteristics among good response and nonresponse subgroup patients

Characteristics	Good response (*N* = 1069) No. (%)	Nonresponse (*N* = 1186) No. (%)	*X* ^2^	*p* value
Age, years, median			0.112	0.738
<56	515 (48.2)	563 (47.5)		
≥56	554 (51.8)	623 (52.5)		
Gender			0.087	0.769
Male	723 (67.6)	809 (68.2)		
Female	346 (32.4)	377 (31.8)		
Clinical T stage			10.307	0.001
cT3	741 (69.3)	746 (62.9)		
cT4	328 (30.7)	440 (37.1)		
Clinical N stage			21.866	<0.001
cN0	238 (22.3)	200 (16.9)		
cN1	474 (44.3)	484 (40.8)		
cN2	357 (33.4)	502 (42.3)		
Clinical TNM stage			10.477	0.001
II	238 (22.3)	200 (16.9)		
III	831 (77.7)	986 (83.1)		
Tumor distance from anus, cm			6.083	0.014
0–5 (low level)	632 (59.1)	640 (54.0)		
5–10 (middle level)	437 (40.9)	546 (46.0)		
Histological grade			5.821	0.054
Low‐differentiated	148 (13.8)	171 (14.4)		
Moderate‐differentiated	771 (72.1)	807 (68.0)		
High‐differentiated	150 (14.1)	208 (17.6)		
nCT cycle, median three cycles			90.914	<0.001
1–3	550 (51.4)	842 (71.0)		
≥4	519 (48.6)	344 (29.0)		
Total cycles, median seven cycles			0.393	0.531
<7	452 (42.3)	517 (43.6)		
≥7	617 (57.7)	669 (56.4)		
Intervals, median 8 weeks			33.983	<0.001
<8	377 (35.3)	562 (47.4)		
≥8	692 (64.7)	624 (52.6)		
Type of radiation			1.990	0.158
3D‐CRT	98 (9.2)	130 (11.0)		
IMRT	971 (90.8)	1056 (89.0)		

Abbreviations: 3D‐CRT, three‐dimensional conformal radiotherapy; IMRT, intensity‐modulated radiotherapy; nCT, neoadjuvant chemotherapy.

### 3.2 Correlation between clinicopathological characteristics and good treatment response

There were no statistically significant differences regarding age, sex, differentiation of the tumors, total cycles of chemotherapy, and the radiation method used between the good response group and nonresponsive group. However, the proportion of low‐level tumors was significantly higher in the good response group than that of nonresponsive group (59.1% vs. 54.0%; *p* = 0.014). The patients with advanced T and N stages in the good response group totaled less than in the nonresponsive group (30.7% vs. 37.1%, *p* = 0.001; 33.4% vs. 42.3%, *p *< 0.001). Moreover, considering the treatment strategies, as compared with in the nonresponsive group, patients who received at least four cycles of nCT were significantly more numerous in the good response group (48.6% vs. 29.0%; *p *< 0.001). Also, patients in the good response group were more likely to be treated with delayed surgery (64.7% vs. 52.6%; *p *< 0.001) (Table 1).

After TME surgery, in the good response group, the proportion of advanced pT and pN stages were lower than those in the nonresponsive group (0% vs. 89.0%, *p *< 0.001; 0% vs. 42.4%, *p *< 0.001). Similarly, patients in the good response group more frequently achieved TRG 0–1 staging (76.8% vs. 28.9%; *p *< 0.001), while those patients who were nonresponders were more likely to have higher positive proportions of circumferential resection margin (CRM) (1.8% vs. 0%; *p *< 0.001), vascular invasion (3.7% vs. 0.9%; *p *< 0.001), and neural invasion (8.5% vs. 0.7%; *p *< 0.001). Remarkably, patients in the nonresponsive group were more likely to have been treated with more cycles of ACT (55.5% vs. 49.4%; *p *= 0.013) (Table S1).

### 3.3 Multivariate logistic regression analysis

**FIGURE 2:**
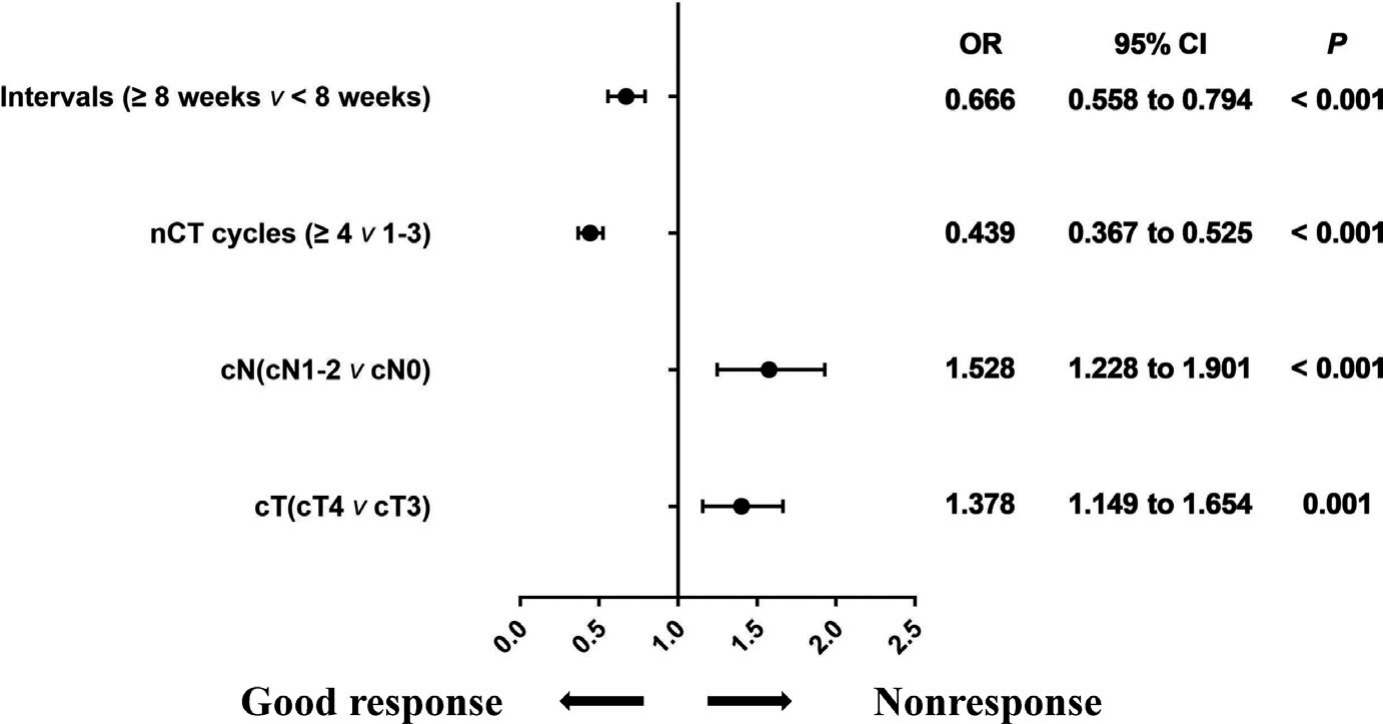
Multivariate logistic regression analysis of the risk for nonresponse for 2255 patients with locally advanced rectal cancer. Abbreviations: CI, confidence interval; nCT, neoadjuvant chemotherapy; OR, odds ratio

We used a multivariate logistic regression model to adjust for the confounded preoperative factors including cT, cN, tumor location, number of cycles of nCT, and whether there was a delay in surgery. As shown in Figure 2, patients who received more cycles of nCT were less likely to achieve a poor response (odds ratio [OR] 0.439, 95% confidence interval [CI]: 0.367–0.525; *p *< 0.001). Similarly, in the good response cohort, more patients underwent delayed surgery after the last fraction of radiation (OR 0.666, 95% CI: 0.558–0.794; *p *< 0.001). Advanced T and N stages were associated with a higher possibility of poor response (OR 1.378, 95% CI: 1.149–1.654; *p *= 0.001 and OR 1.528, 95% CI: 1.228–1.901; *p *< 0.001).

### 3.4 Correlation between different treatment strategies and good treatment response

When combining the number of cycles of the nCT (1–3 vs. ≥4) and interval to surgery (<8 weeks vs. ≥8 weeks), patients could be divided in four subgroups: less cycles and no delayed surgery (LN) (*n* = 675 patients), less cycles and delayed surgery (LD) (*n* = 717 patients), more cycles and no delayed surgery (MN) (*n *= 264 patients), and more cycles and delayed surgery (MD) (*n *= 599 patients). Intergroup comparisons of clinicopathological factors, such as sex and tumor location revealed no significant differences. Nevertheless, patients in the MN and MD groups were much younger than those in the LN and LD groups (<56 vs. ≥56) (55.3% and 52.9% vs. 47.7% and 40.9%; *p *< 0.001). Similarly, in the MN and MD groups, patients more frequently presented with pathological poor differentiation than did those in the LN and LD groups (20.8% and 19.2% vs. 11.6% and 9.9%; *p *< 0.001). Also, as compared with the patients in the LN and LD subgroups, patients in the MN and MD groups were more likely to have advanced cT stages (39.8% and 36.9% vs. 31.9% and 31.7%, *p *= 0.026), and those patients in the LD and MD groups were more likely to have higher proportions of advanced cN stages than those in the LN group (82.0% and 85.0% vs. 75.1%, *p *< 0.001). Moreover, the proportion of patients who received IMRT was significantly higher in the MN and MD groups than in the LN and LD subgroups (97.3% and 98.8% vs. 78.2% and 90.7%; *p *< 0.001). Especially, when compared with the response to nCRT, the proportion of patients with a good response in the MD group (64.9%) was significantly higher than those in LN, LD, and MN subgroups (36.6%, 42.3%, and 49.2%; *p *< 0.001) (Table 2).

**TABLE 2:** Preoperative clinicopathological characteristics among different treatment strategies

Characteristics	nCT cycles (<4 cycle vs. ≥4 cycle); time to surgery (<8 weeks vs. ≥8 weeks)				
	<4 & <8 (LN) (*N* = 675) No. (%)	<4 & ≥8 (LD) (*N* = 717) No. (%)	≥4 & <8 (MN) (*N* = 264) No. (%)	≥4 & ≥8 (MD) (*N* = 599) No. (%)	*p* value
Age, years, median					<0.001
<56	322 (47.7)	293 (40.9)	146 (55.3)	317 (52.9)	
≥56	353 (52.3)	424 (59.1)	118 (44.7)	282 (47.1)	
Gender					0.161
Male	448 (66.4)	475 (66.2)	181 (68.6)	428 (71.5)	
Female	227 (33.6)	242 (33.8)	83 (31.4)	171 (28.5)	
Clinical T stage					0.026
cT3	460 (68.1)	490 (68.3)	159 (60.2)	378 (63.1)	
cT4	215 (31.9)	227 (31.7)	105 (39.8)	221 (36.9)	
Clinical N stage					<0.001
cN0	168 (24.9)	129 (18.0)	51 (19.3)	90 (15.0)	
cN1–2	507 (75.1)	588 (82.0)	213 (80.7)	509 (85.0)	
Tumor distance from anus, cm					0.879
0–5	377 (55.9)	403 (56.2)	146 (55.3)	346 (57.8)	
5–10	298 (44.1)	314 (43.8)	118 (44.7)	253 (42.2)	
Histological grade					<0.001
Low‐differentiated	78 (11.6)	71 (9.9)	55 (20.8)	115 (19.2)	
Moderate‐ and high‐differentiated	597 (88.4)	646 (90.1)	209 (79.2)	484 (80.8)	
Type of radiation					<0.001
IMRT	528 (78.2)	650 (90.7)	257 (97.3)	592 (98.8)	
3D‐CRT	147 (21.8)	67 (9.3)	7 (2.7)	7 (1.2)	
Response to nCRT					<0.001
Good	247 (36.6)	303 (42.3)	130 (49.2)	389 (64.9)	
None	428 (63.4)	414 (57.7)	134 (50.8)	210 (35.1)	

Abbreviations: 3D‐CRT, three‐dimensional conformal radiotherapy; IMRT, intensity‐modulated radiotherapy; LD, less cycles and delayed surgery; LN, less cycles and no delayed surgery; MD, more cycles and delayed surgery; MN, more cycles and no delayed surgery; nCRT, neoadjuvant chemoradiotherapy.

By comparing the mean number of cycles of the nCT, patients in the MN and MD groups appeared to receive more cycles of induction chemotherapy cycles than those in the LN and LD groups (1.7 [1.7 ± 0.9] and 1.7 [1.7 ± 1.0], vs. 1.0 [1.0 ± 0.0] and 1.1 [1.1 ± 0.3]; *p *< 0.001), while similar results were found among patients who were delivered cycles of concurrent chemotherapy (2.9 [2.9 ± 0.9] and 2.8 [2.8 ± 0.9] vs. 2.1 [2.1 ± 0.5] and 2.1 [2.1 ± 0.5]; *p *< 0.001). In terms of consolidation chemotherapy cycles, we found that, in the MD group, the mean number of cycles delivered to patients was 2.1 (2.1 ± 0.9), which was higher than in the MN, LN, and LD groups (MN: 1.4 [1.4 ± 0.6]; LN: 1.0 [1.0 ± 0.0]; and LD: 1.1 [1.1 ± 0.3]; *p *< 0.001). Similarly, the total of nCT cycles in the MD group was 5.4 (5.4 ± 1.5), which was significantly more than those in the other three subgroups (MN: 4.9 [4.9 ± 1.1]; LN: 2.3 [2.3 ± 0.5]; LD: 2.4 [2.4 ± 0.6]; *p *< 0.001) (Table S2).

### 3.5 Survival analysis

**FIGURE 3:**
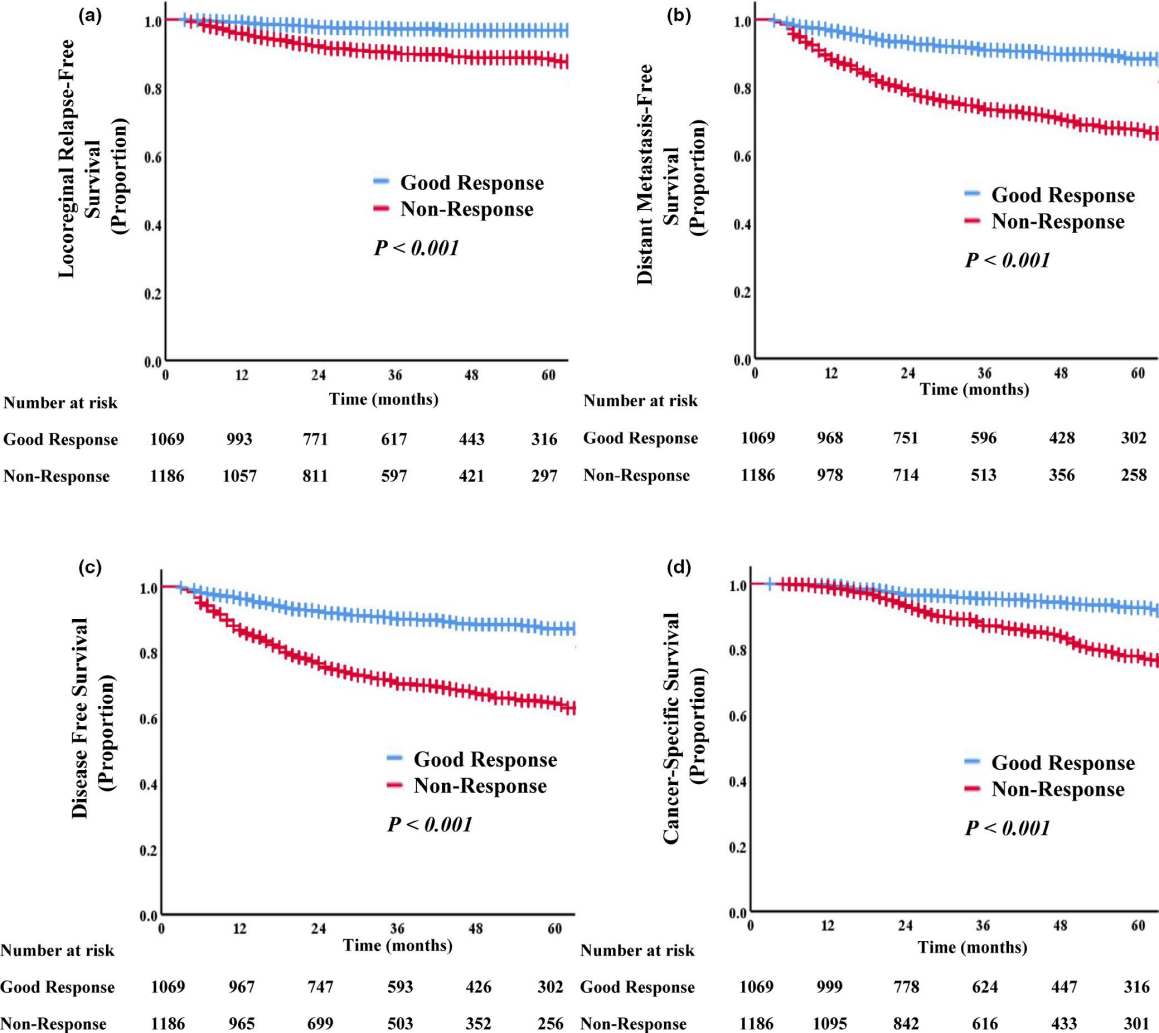
Kaplan–Meier curve analysis of LRFS (A), DMFS (B), DFS (C), and CSS (D), comparing patients with good responses and nonresponses. Abbreviations: CSS, cancer‐specific survival; DFS, disease‐free survival; DMFS, distant metastasis‐free survival; LRFS, locoregional relapse‐free survival

The Kaplan–Meier survival curve analysis confirmed that, among patients in the responsive group, the 3‐year CSS, LRFS, DMFS, and DFS rates were significantly better than those in the nonresponsive group (CSS: 95.3% vs. 87.3%, *p *< 0.001; LRFS: 97.2% vs. 90.3%, *p* < 0.001; DMFS: 91.1% vs. 73.5%, *p *< 0.001; DFS: 90.1% vs. 70.4%, *p *< 0.001) (Figure 3).

**FIGURE 4:**
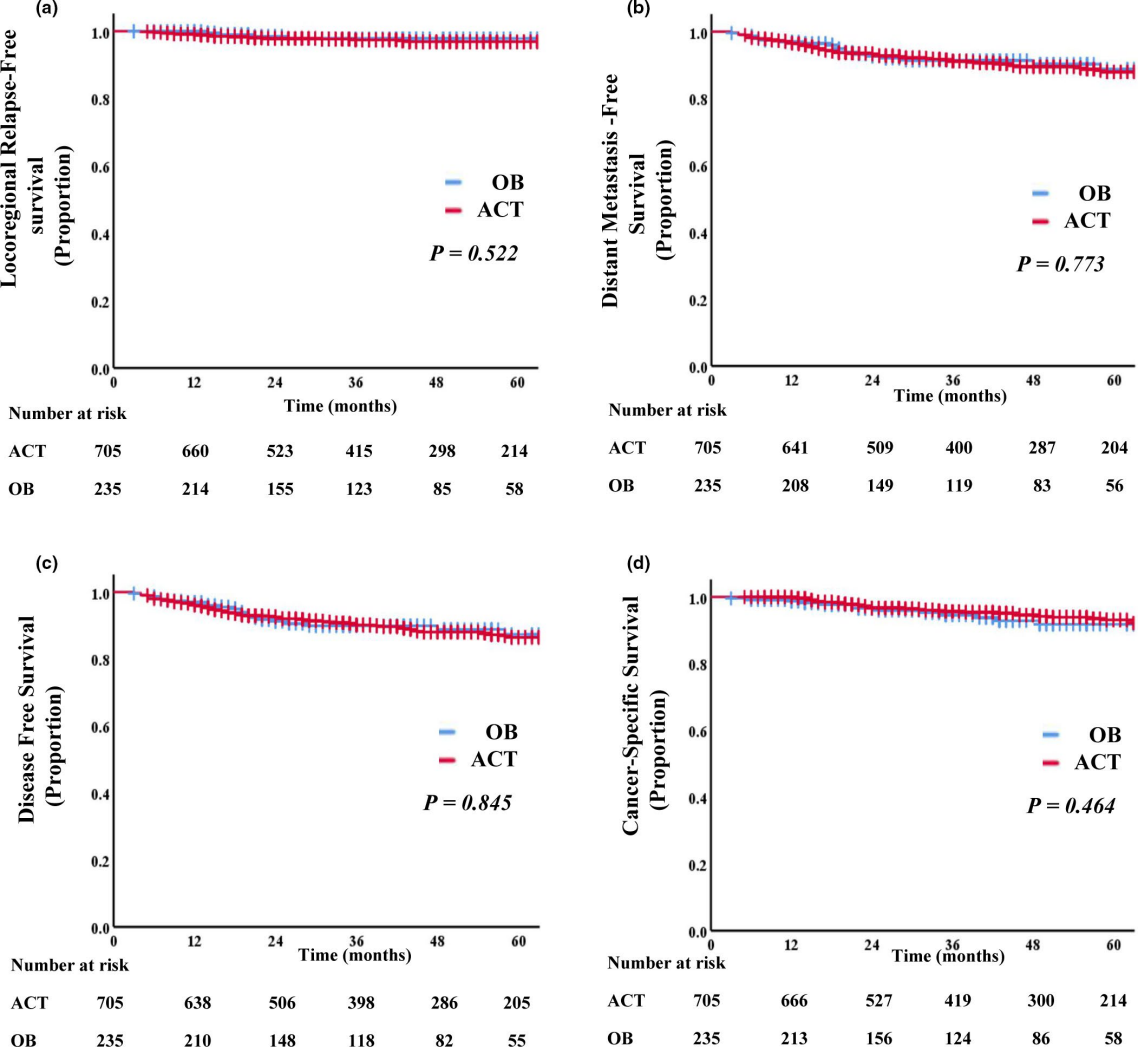
Kaplan–Meier curve analysis of LRFS (A), DMFS (B), DFS (C), and CSS (D), comparing the ypT0–2N0M0 patients without or with adjuvant chemotherapy after PSM (1:3). Abbreviations: ACT, adjuvant chemotherapy; CSS, cancer‐specific survival; DFS, disease‐free survival; DMFS, distant metastasis‐free survival; LRFS, locoregional relapse‐free survival; OB, observation

The baseline clinicopathological profiles of the good responders are shown in Table 3. Before PSM, the number of good responders was 1069. As compared with those treated with ACT, there were more men in the OB group (74% vs. 65.8%; *p* = 0.017) and the proportion of the patients with clinical II stage disease in the OB group was greater than that in the ACT group (28.1% vs. 20.6%; *p *= 0.015). Also, less cases with a low‐level rectal cancer were seen in the ACT group (57.4% vs. 65.1%; *p* = 0.035). After PSM (Figure S1), the numbers of the patients in the OB and ACT groups were 235 and 705, respectively, and the baseline clinicopathological characteristics were balanced in these two subgroups. The survival analysis revealed that no significant difference existed regarding 3‐year CSS (93.8% vs. 95.5%; *p *= 0.464), DFS (88.9% vs. 90.2%; *p *= 0.845), LRFS (97.4% vs. 97.8%; *p *= 0.522), or DMFS (90.3% vs. 91.2%; *p* = 0.773) between the OB and ACT groups (Figure 4).

**TABLE 3:** Patients characteristics before and after PSM among yp0–I stage subgroup patients

Characteristics	Before matching			After matching		
	OB no. (%) (*N* = 235)	ACT no. (%) (*N* = 834)	*p* value	OB no. (%) (*N* = 235)	ACT no. (%) (*N* = 705)	*p* value
Age, years, median			0.071			0.472
<56	101 (43.0)	414 (49.6)		101 (43.0)	322 (45.7)	
≥56	134 (57.0)	420 (50.4)		134 (57.0)	383 (54.3)	
Gender			0.017			0.475
Male	174 (74.0)	549 (65.8)		174 (74.0)	505 (71.6)	
Female	61 (26.0)	285 (34.2)		61 (26.0)	200 (28.4)	
Clinical T stage			0.736			0.902
cT3	165 (70.2)	576 (69.1)		165 (70.2)	492 (69.8)	
cT4	70 (29.8)	258 (30.9)		70 (29.8)	213 (30.2)	
Clinical N stage			0.104			0.183
cN0	66 (28.1)	172 (20.6)		66 (28.1)	163 (23.1)	
cN1	94 (40.0)	380 (45.6)		94 (40.0)	297 (42.1)	
cN2	75 (31.9)	282 (33.8)		75 (31.9)	245 (34.8)	
Clinical TNM stage			0.015			0.125
II	66 (28.1)	172 (20.6)		66 (28.1)	163 (23.1)	
III	169 (71.9)	662 (79.4)		169 (71.9)	542 (76.9)	
Tumor distance from anus, cm			0.035			0.371
0–5	153 (65.1)	479 (57.4)		153 (65.1)	436 (61.8)	
5–10	82 (34.9)	355 (42.6)		82 (34.9)	269 (38.2)	
Histological grade			0.734			0.727
High‐differentiated	36 (15.3)	112 (13.4)		36 (15.3)	104 (14.7)	
Moderate‐differentiated	165 (70.2)	606 (72.7)		165 (70.2)	513 (72.8)	
Low‐differentiated	34 (14.5)	116 (13.9)		34 (14.5)	88 (12.5)	
nCT cycle, median three cycles			0.872			0.910
1–3	122 (51.9)	428 (51.3)		122 (51.9)	363 (51.5)	
≥4	113 (48.1)	406 (48.7)		113 (48.1)	342 (48.5)	
Time to surgery, median 8 weeks			0.093			0.494
<8	72 (30.6)	305 (36.6)		72 (30.6)	233 (33.0)	
≥8	163 (69.4)	529 (63.4)		163 (69.4)	472 (67.0)	
Type of radiation			0.530			0.701
IMRT	211 (89.8)	760 (91.1)		211 (89.8)	639 (90.6)	
3D‐CRT	24 (10.2)	74 (8.9)		24 (10.2)	66 (9.4)	

Abbreviations: 3D‐CRT, three‐dimensional conformal radiotherapy; ACT, adjuvant chemotherapy; IMRT, intensity‐modulated radiotherapy; nCT, neoadjuvant chemotherapy; OB, observation.

## 4 DISCUSSION

In this study, we found that patients with a good response to nCRT (ypT0–2N0M0) had a significantly better prognosis than that of patients with nonresponse (ypT3–4N0M0 or ypT0–4N+M0). Furthermore, when we analyzed the correlation of clinicopathological characteristics and good treatment response, the logistic regression model showed that less advanced cT and cN stages, more nCT cycles (≥4), and delayed surgery (≥8 weeks) were independent predictors of a good response to nCRT. Especially, the group of patients treated with both more cycles and delayed surgery attained the greatest proportion of good responses. Meanwhile, we evaluated the necessity of ACT for ypT0–2N0 rectal cancer and the results showed that adding postoperative ACT had no survival benefit.

Similar with our study, accumulated research has demonstrated that postoperative pathological stage after nCRT was strongly associated with prognosis among patients with LARC, which could affect subsequent treatment selections.^5^, ^6^, ^8^, ^10^ For example, the ‘wait‐and‐watch’ policy may be accepted if pCR could be predicted after nCRT but before TME surgery,^6^ while, for ypT1–2N0 LARC patients in the middle and low position, local excision could be selectively deployed to preserve the organ.^9^, ^10^ Thus, many previous studies have evaluated the correlation factor and demonstrated their capacities to predict good response to nCRT.^8^, ^11^, ^13^, ^18^ For instance, less advanced tumor T and N stages significantly related to a good response after nCRT have been reported,^8^, ^11^ similar to our study.

Nevertheless, different from the previous studies, given that the patients in our study were treated using several treatment strategies, we also explored the best pairing of the number of nCT cycles and the waiting time for surgery. A phase II study from Royal Marsden Hospital reported that adding induction chemotherapy prior to CRT could achieve a pCR rate at 20% without increasing the level of toxicity,^25^ while another study from the same hospital revealed that induction chemotherapy could improve the MRI‐EMVI status, which was an independent predictor of a good response to nCRT.^8^ Similarly, adding consolidation chemotherapy after CRT resulted in more good responses.^15^, ^17^ In our study, delivering more nCT cycles (≥4) was an independent predictor of a good response. The fact that most of the patients with more nCT cycles were administered induction or consolidation chemotherapy or both may explain why these patients were prone to achieving a better response.

Another independent predictor we found in our study was that delayed surgery was an independent predictor of a good response. Previous data have suggested that delaying surgery after nCRT was correlated with poorer survival.^26^, ^27^ However, similar to our study, a large‐scale meta‐analysis that enrolled 13 clinical trials indicated that a longer waiting interval (>6–8 weeks) was useful in increasing the rate of pCR without increasing the complication rates.^28^ The possible reason for this was that the degree of tumor regression has been reported to be dependent on the time from the end of radiation.^28^

During further analysis, we found that the treatment strategy of combining more chemotherapy cycles and delaying surgery led to the highest proportion of good responses in the whole cohort. Similar to our results, two prospective studies from MSKCC and CAO/ARO/AIO‐12 also reported that adding more cycles of chemotherapy before radical surgery and delaying surgery resulted in more good responses.^15^, ^17^ Recently, the RAPIDO randomized trial reported that, with this combination strategy, doubling of the pCR rates (from 14% to 28%; *p* < 0.0001) could be achieved.^14^ In addition, it was notable that, in our study, patients treated with this combination strategy more frequently had advanced T and N stages, poor differentiation, and younger age, implying that this strategy may be an optimal option for the patients with worse clinicopathological characteristics, but with a better tolerance to intensive chemotherapy. Additionally, the type of radiation therapy delivered to these patients was almost always IMRT. Compared with 3D‐CRT, IMRT has the advantage of decreasing the rate of radiation enteritis.^29^ Thus, more powerful and cycles of chemotherapy could be delivered.

Among patients who achieved a good response, we analyzed the necessity of ACT, as this remains controversial. Results from some studies have failed to support the survival benefits of giving ACT. For example, Liao et al. retrospective study showed that adding ACT had no effect on the 5‐year DFS rate,^22^ and a recent study showed the postoperative XELOX without preoperative chemoradiation is effective for rectal cancer and provides adequate 3‐year DFS aspect.^30^ However, a different study from a large‐scale database supported delivering ACT to patients with pCR to obtain OS benefits.^19^ Nevertheless, in previous studies, either the data of nCT regimens and cycles or regarding the intervals from the last fraction to surgery were not complete or the characteristics among the groups were unbalanced.^5^, ^7^, ^19^, ^21^, ^22^ Thus, it was difficult to draw an exact conclusion. In our study, we included and balanced the preoperative clinicopathological characteristics and analyzed detailed survival outcomes information and proved that ACT did not improve CSS, DFS, LRFS, and DMFS.

In our study, there were some advantages and disadvantages. First, considering that the pCR patients were the main study objects in previous studies,^12^, ^13^, ^18^, ^20^ we also included ypT1–2N0 patients and our study participants also showed a good prognosis and, more importantly, were potential candidates for local excision selection.^9^ Moreover, to the best of our knowledge, this was the largest‐scale study to analyze the correlation of clinicopathological characteristics including different nCT strategies and the intervals to surgery and good response; furthermore, we attained an answer as to whether ACT was necessary for these patients. However, based on the retrospective nature, data on magnetic resonance imaging parameters such as EMVI, MRF, tumor size, and the blood sample and biopsy specimen were incomplete. Therefore, the genomics analysis was not conducted in our research, although some biologically predictive characteristics have been explored in previous studies.^11^, ^25^, ^31^, ^32^, ^33^ Besides, the chemotherapy regimens in this study were not consistent, and the response assessment might influence the number of chemotherapy cycles during treatment. Therefore, we reduced this limitation by enrolling a considerable number of patients and conducted PSM analysis to balance the differences.

## 5 CONCLUSIONS

Our study demonstrated that good responders to nCRT in a LARC population experienced significantly better 3‐year CSS, LRFS, DMFS, and DFS rates than the nonresponsive patients. We also identified clinicopathological predictive factors to good response; our results showed that less advanced cT and cN stages, more nCT cycles (≥4), and delayed surgery (≥8 weeks) were significantly associated with a good response to nCRT. Especially, the combination of more nCT cycles and a delay in surgery achieved the highest proportion of good responses. Further analysis demonstrated that ACT in the good responders was not recommended.

## CONFLICT OF INTEREST

The authors declare that they have no competing interests.

## ETHICAL STATEMENT

The protocol was approved by the ethics committee of the Sixth Affiliated Hospital, Sun Yat‐sen University (2021ZSLYEC‐051).

## Supporting information


**Figure S1**.

**Table S1**.

**Table S2**.

## Data Availability

The datasets used during the current study are available from the corresponding author upon reasonable request.
