# Supplementary figures and images for: Associations between clinical characteristics and tumor response to neoadjuvant chemoradiotherapy in rectal cancer
Source: Cancer Med. 2021 Jun 15;10(14):4832–43. doi: 10.1002/cam4.4051 (PMC8290248; doi:10.1002/cam4.4051)

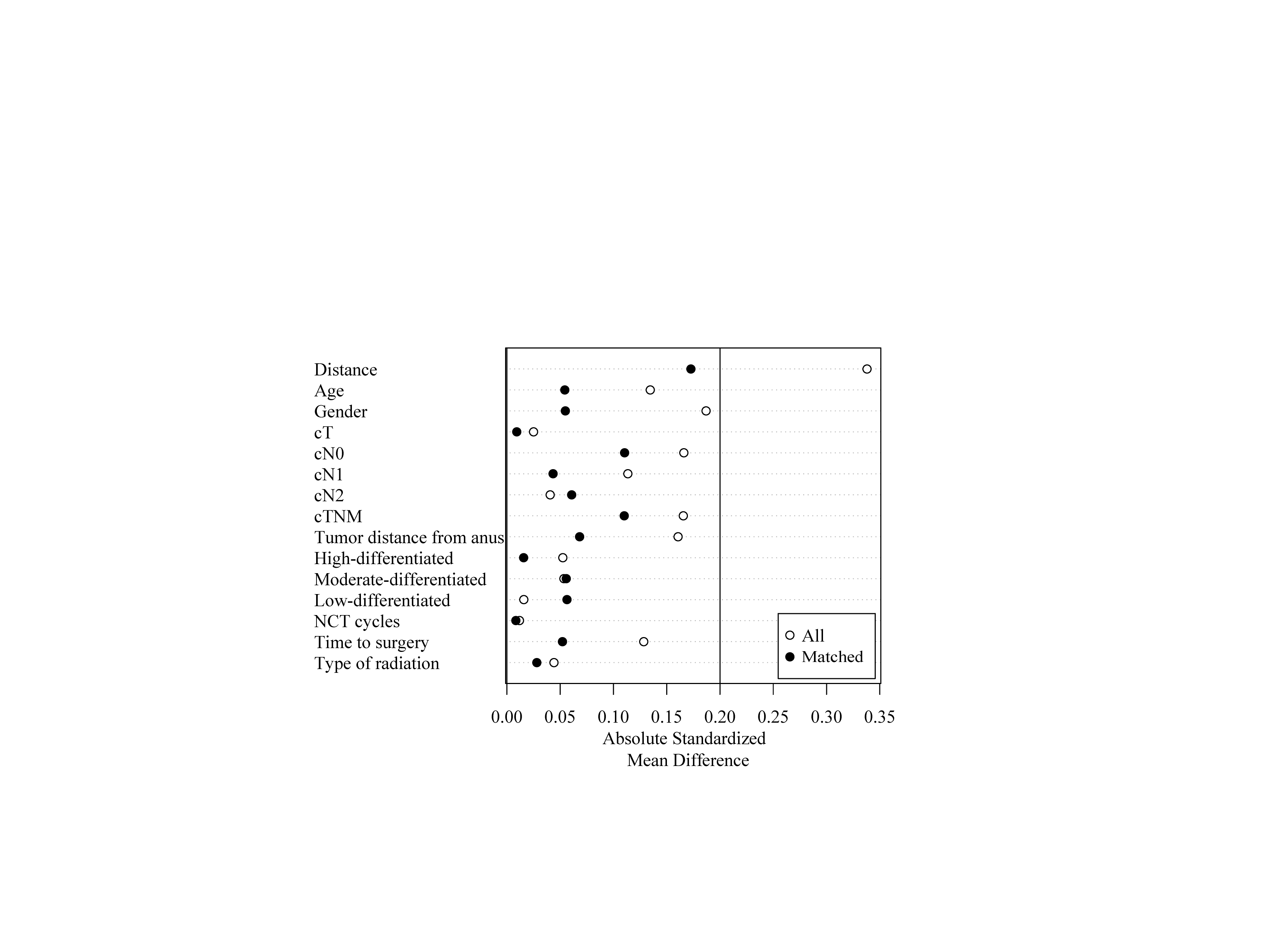

Supplement: Supplementary file 1 — Figure S1. [file CAM4-10-4832-s003.tif]
