# Supplementary material for: Associations between clinical characteristics and tumor response to neoadjuvant chemoradiotherapy in rectal cancer
Source: Cancer Med. 2021 Jun 15;10(14):4832–43. doi: 10.1002/cam4.4051 (PMC8290248; doi:10.1002/cam4.4051)
Supplement: Supplementary file 2 — Table S1. [file CAM4-10-4832-s002.docx]

**Table S1.** Postoperative clinicopathological characteristics among good response and nonresponse subgroup patients.

| Characteristics | Good response  (N = 1069) No. (%) | Nonresponse  (N = 1186) No. (%) | *X^2^* | *P* value |
| --- | --- | --- | --- | --- |
| Pathologically T stage |  |  | 1786.9 | < 0.001 |
| pT0-2 | 1069 (100.0) | 131 (11.0) |  |  |
| pT3-4 | 0 (0.0) | 1055 (89.0) |  |  |
| Pathologically N stage |  |  | 583.544 | < 0.001 |
| pN0 | 1069 (100.0) | 683 (57.6) |  |  |
| pN1-2 | 0 (0.0) | 503 (42.4) |  |  |
| TRG |  |  | 518.787 | < 0.001 |
| 0-1 | 821 (76.8) | 343 (28.9) |  |  |
| 2 | 223 (20.9) | 717 (60.5) |  |  |
| 3 | 25 (2.3) | 126 (10.6) |  |  |
| Vascular invasion |  |  | 18.517 | < 0.001 |
| negative | 1059 (99.1) | 1142 (96.3) |  |  |
| positive | 10 (0.9) | 44 (3.7) |  |  |
| Neural invasion |  |  | 73.745 | < 0.001 |
| negative | 1061 (99.3) | 1085 (91.5) |  |  |
| positive | 8 (0.7) | 101 (8.5) |  |  |
| Surgical margin |  |  | 4.517 | 0.064 |
| negative | 1069 (100.0) | 1181 (99.6) |  |  |
| positive | 0 (0.0) | 5 (0.4) |  |  |
| Circumferential resection margin, mm |  |  | 19.106 | < 0.001 |
| ≤ 1 | 1069 (100.0) | 1165 (98.2) |  |  |
| > 1 | 0 (0.0) | 21(1.8) |  |  |
| ACT cycle, median 3 cycles |  |  | 8.704 | 0.013 |
| 0 | 235 (22.0) | 220 (18.5) |  |  |
| 1-3 | 306 (28.6) | 308 (26.0) |  |  |
| 4-6 | 528 (49.4) | 658 (55.5) |  |  |

Abbreviations: TRG, tumor regression grade; ACT, adjuvant chemotherapy.
