# Supplementary material for: Associations between clinical characteristics and tumor response to neoadjuvant chemoradiotherapy in rectal cancer
Source: Cancer Med. 2021 Jun 15;10(14):4832–43. doi: 10.1002/cam4.4051 (PMC8290248; doi:10.1002/cam4.4051)
Supplement: Supplementary file 3 — Table S2. [file CAM4-10-4832-s001.docx]

**Table S2.** Induction, concurrent, consolidation, and total neoadjuvant chemotherapy cycles, in 2255 patients with locally advanced rectal cancer.

|  | NCT cycles ( < 4 cycle vs. ≥ 4 cycle ); Time to surgery ( < 8 weeks vs. ≥ 8 weeks) | | | | | | | |  |
| --- | --- | --- | --- | --- | --- | --- | --- | --- | --- |
| Chemotherapy | LN (< 4 & < 8) (N = 675) | | LD (< 4 & ≥ 8) (N = 717) | | MN ( ≥ 4 & < 8) (N = 264) | | MD ( ≥ 4 & ≥ 8) (N = 599) | | *P* value |
|  | patients  No. (%) | cycles  Mean (SD) | patients  No. (%) | cycles  Mean (SD) | patients  No. (%) | cycles  Mean (SD) | patients  No. (%) | cycles  Mean (SD) |  |
| Induction |  |  |  |  |  |  |  |  | < 0.001 |
| No | 644 (95.4) |  | 697 (97.2) |  | 84 (31.8) |  | 194 (32.4) |  |  |
| Yes | 31 (4.6) | 1.00 (1.0 ± 0.0) | 20 (2.8) | 1.1 (1.1 ± 0.3) | 180 (68.2) | 1.7 (1.7 ± 0.9) | 405 (67.6) | 1.7 (1.7 ± 1.0) |  |
| Concurrent |  |  |  |  |  |  |  |  | < 0.001 |
| No | 0 (0.0) |  | 0 (0.0) |  | 0 (0.0) |  | 0 (0.0) |  |  |
| Yes | 675 (100.0) | 2.1 (2.1 ± 0.5) | 717 (100.0) | 2.1 (2.1 ± 0.5) | 264 (100.0) | 2.9 (2.9 ± 0.9) | 599 (100.0) | 2.8 (2.8 ± 0.9) |  |
| Consolidation |  |  |  |  |  |  |  |  | < 0.001 |
| No | 583 (86.4) |  | 556 (77.5) |  | 114 (43.2) |  | 237 (39.6) |  |  |
| Yes | 92 (13.6) | 1.0 (1.0 ± 0.0) | 161 (22.5) | 1.1 (1.1 ± 0.3) | 150 (56.8) | 1.4 (1.4 ± 0.6) | 362 (60.4) | 2.1 (2.1 ± 0.9) |  |
| Total nCT | 675 | 2.3 (2.3 ± 0.5) | 717 | 2.4 (2.4 ± 0.6) | 264 | 4.9 (4.9 ± 1.1) | 599 | 5.4 (5.4 ± 1.5) | < 0.001 |

Abbreviations: LN,less cycles and no delayed surgery; LD, less cycles and delayed surgery; MN, more cycles and no delayed surgery; MD, more cycles and delayed surgery; nCT, neoadjuvant chemotherapy; SD, standard deviation.
